# Nanoscience Supporting the Research on the Negative Electrodes of Li-Ion Batteries

**DOI:** 10.3390/nano5042279

**Published:** 2015-12-16

**Authors:** Alain Mauger, Christian M. Julien

**Affiliations:** 1 Sorbonne Universités, UPMC Université Paris6, Institut de Minéralogie et Physique de la Matière Condensée (IMPMC), 4 place Jussieu, Paris 75005, France; E-Mail: alain.mauger@impmc.jussieu.fr; 2 Sorbonne Universités, UPMC Université Paris6, Physicochimie des Electrolytes et Nanosystèmes Interfaciaux (PHENIX), UMR 8234, 4 place Jussieu, Paris 75005, France

**Keywords:** nano-materials, negative electrodes, Li-ion batteries

## Abstract

Many efforts are currently made to increase the limited capacity of Li-ion batteries using carbonaceous anodes. The way to reach this goal is to move to nano-structured material because the larger surface to volume ratio of particles and the reduction of the electron and Li path length implies a larger specific capacity. Additionally, nano-particles can accommodate such a dilatation/contraction during cycling, resulting in a calendar life compatible with a commercial use. In this review attention is focused on carbon, silicon, and Li_4_Ti_5_O_12_ materials, because they are the most promising for applications.

## 1. Introduction

Since they became commercialized, all the lithium-ion batteries are equipped with graphitic carbon anodes. The graphite can reversibly absorb lithium up to the concentration LiC_6_. One advantage of using carbon is its very good electrical conductivity, and good ionic conductivity. It must be protected against side reactions with the electrolyte, but chemists have found solvents such as ethylene carbonate that allow the formation of a stable solid-electrolyte interface (SEI). However, the theoretical capacity associated to the cycles between C and LiC_6_ is 372 mAh·g^−1^. Therefore, many efforts in research are currently made to increase this limited capacity. The way to reach this goal is to move to nano-structured anodes for three reasons: (a) when the size of the active particles is decreased to the nano range, the larger surface to volume ratio implies a larger specific capacity, and a larger contact area with the electrolyte leading to high lithium ion flux across the electrode/electrolyte interface; (b) the reduction of the size of the particles implies a reduction of the length of the electron and Li path inside the particles. This migration distance inside the particle is the limiting factor for conductivity, because after the electron has reached the surface of a particle it is driven to the current collector in a conductive medium, while the Li^+^ ion is driven to the counter-electrode through an electrolyte which has a much larger ionic conductivity than Si. Therefore, the overall lithium diffusion and electrical conductivity of the assembly of Si particles are improved by the reduction of the size of Si particles to the nano-range, leading to batteries with enhanced power capability; and (c) the intercalation of lithium is accompanied with a dilatation of the particles, which may result in the formation of cracks, or even pulverization when the particle size is in the micron range. Furthermore, nano-particles can accommodate such a dilatation/contraction during cycling, resulting in a calendar life compatible with a commercial use. The first idea is to keep carbon under nano-size in form, such as nanotubes, or graphene, but attempts are also currently made to replace carbon by other materials with higher theoretical capacity. Among them, silicon is considered as most promising, because it has a very large theoretical capacity, and because we can benefit from the silicon technology in electronics to synthesize nano-structured particles of different shapes. On the other hand, even with the size of Si particles is reduced to the nano-range, the lithiation/delithiation must be a slow process to limit the mechanical stress, meaning that the power available with batteries equipped with a Si-based anode is small. Therefore, other efforts have been made to obtain Li-ion batteries that have much higher power, even if their energy density is much smaller. Li_4_Ti_5_O_12_ is considered as the most promising anode with this respect. We do not intend to review here all the works devoted to anode materials. We only report recent results and progress that illustrate the outstanding improvements in the electrochemical performance of Li-ion batteries, resulting from the reduction of the size of the active particles to the nano-scale. More exhaustive reviews devoted to anodes can be found in review papers and books [1,2]. Additionally, attention is focused here on the carbon, silicon, and Li_4_Ti_5_O_12_ materials, because they are the most promising for applications. Many other oxides are currently under investigation in laboratories, and we only guide the reader to the recent book [2] that reviews the state of the art on them.

## 2. Nano-Structured Carbon

The quantum confinement modifies the electronic and structural properties, which is beneficial to the storage capacity by removing the limitation of the capacity to 372 mAh·g^−1^ [3,4,5,6,7,8]. The increase of the number of storage sites available for the lithium ions in small closed spaces in nano-structured carbon is another reason invoked to explain the large capacities that have been observed [9,10,11]. Carbon nano-rings with 20 nm outer diameters and 3.5-nm wall thickness deliver a capacity 1200 mAh·g^−1^ over hundreds of cycles at the current density of 0.4 A·g^−1^. At the higher current rates of 45 A·g^−1^, the capacity is still 500 mAh·g^−1^ [12].

Carbon nanotubes, especially single wall carbon nanotubes (SWCNTs) are promising, as their reversible capacity is estimated to be 1116 mAh·g^−1^ in stoichiometric LiC_2_ owing to the intercalation of lithium into stable sites located on the surface of pseudo-graphitic layers and inside the central tube, as well [13,14,15,16]. Indeed, a purified single-wall carbon nanotube (SWCNT) obtained by laser vaporization delivered a capacity larger than 1050 mAh·g^−1^ [17]. The best result with multi-wall carbon nanotubes (MWCNT) has been obtained through directly applying a 10 nm thick layer of Al_2_O_3_ by atomic layer deposition (ALD) on the pre-fabricated electrodes made from bare powders. The resultant MWCNT anode demonstrated a stable capacity of 1100 mAh·g^−1^ in 50 charge-discharge cycles at the current rate of 372 mA·g^−1^ [18]. The Al_2_O_3_ coating effectively blocks electron tunneling to the adsorbed EC molecules of the electrolyte and, thus, decreases the decomposition of the electrolyte [19]. Note that bad results are obtained when coating bare electrode materials via ALD and then fabricating the coated materials into electrodes, because Al_2_O_3_ is insulating and inhibits the electron conduction path between the anode and the current collector [20]. Note, however, that these results are obtained at the laboratory scale and are far from the results obtained with commercial products. Commercial MWCNTs have a typical capacity close to 250 mAh·g^−1^. After purification, the capacity raises to a 400 mAh·g^−1^. Moreover, the MWCNTs show a tendency to suffer from Li-induced embrittlement [21], because their close structure does not allow expansion of graphene sheets in the c-axis or radial direction, as in graphite, when Li is inserted, thus inducing large stresses during cycling. Despite the promising results we have reported, the carbon nanotubes have not found a place in the industry of the lithium-ion batteries, essentially because of their cost and the difficulty to prepare them free of any large structural defects and high voltage hysteresis.

**Figure 1:**
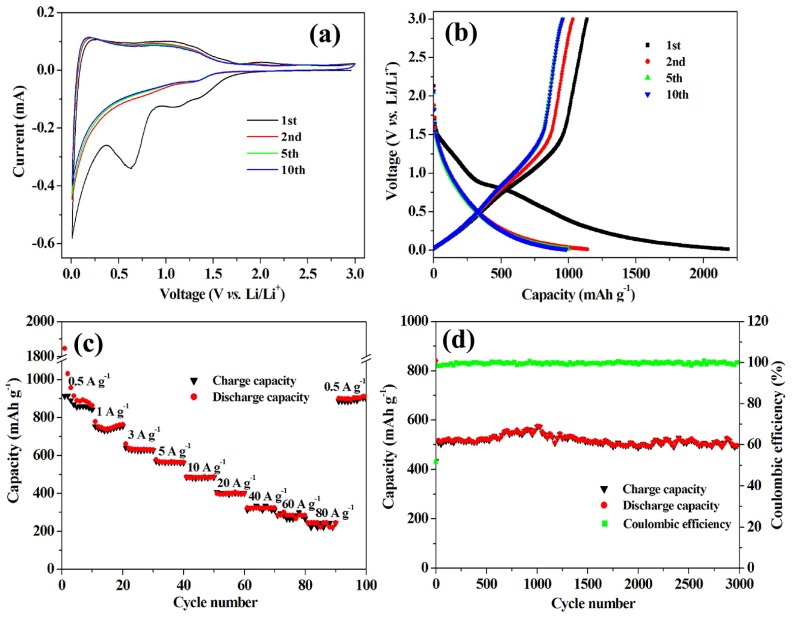
Electrochemical performance of the doped hierarchically porous graphene electrodes. Reproduced with permission from [30]. Copyright The American Chemical Society, 2013. (**a**) Cyclic voltammograms at a scan rate of 0.1 mV·s^−1^; (**b**) charge-discharge curves at 0.1 A·g^−1^; (**c**) capacity over cycling at different current densities; and (**d**) cycling and Coulombic efficiency at current density of 5 A·g^−1^.

Graphene looks more attractive, because of its very good conductivity and mechanical strength [22,23,24,25,26]. The theoretical capacity is much larger than that of graphite, namely 780 mAh·g^−1^ if Li ions can be absorbed on both sites up to the stoichiometric Li_2_C_6_, and 1116 mAh·g^−1^ if the Li ions can be trapped at the benzene rings in a covalent bonding up to LiC_2_ stoichiometry [23,27,28]. The main difficulty with graphene is its tendency to agglomerate, which is a major cause of the aging upon cycling. To avoid this re-stacking of the graphene sheets, the graphene can be doped. Nitrogen, which has five valence electrons and has a comparable atomic size with C, is the most popular dopant, forming a strong covalent C-N bond breaking the charge neutrality on C. This doping generates a disorder in the honeycomb lattice of pristine graphene, which may help in the prevention against re-stacking of the graphene sheets, and it donates more electrons to the carbon network, thus increasing the electrical conductivity [29]. An outstanding result has been obtained with a doped hierarchically-porous graphene (DHPG) electrode using graphene oxide, sulfonated polystyrene spheres (S-PS), and poly(vinyl pyrrolidone) (PVP) as precursors through *in situ* preparation in nickel foam oxide [30]. Electrochemical performance of the doped hierarchically-porous graphene electrodes is shown in Figure 1. During the pyrolysis of the precursor, the graphene was doped *in situ* by nitrogen atoms from PVP and sulfur atoms from S-PS. This electrode delivered a high-power density of 116 kW kg^−1^, whereas the energy density is left as high as 322 Wh·kg^−1^ at 80 A·g^−1^ (about 10 s to full charge) with a good capacity retention for 3000 cycles with high capacities in the temperature range 20–55 °C. This last result shows that doping is beneficial to the performance of graphene as an anode. In a different approach, composites of graphene with nanoparticles of another electroactive anode material have been investigated, since the grafting of the nanoparticles on the graphene sheets prevents the re-stacking. In addition, the elastically strong, flexible, and conductive graphene can accommodate the volume changes suffered by the particles upon cycling, thus benefiting the structural stabilization of the nanoparticles and the cycling life. Actually, doped graphene can be used, thus combining the two effects of doping and grafting. For instance, Co_3_Sn_2_/Co nanoparticles grafted on N-doped graphene tested as an anode delivered capacities of 1600 mAh·g^−1^ and 800 mAh·g^−1^ almost constant between the second and the 100th cycle in the voltage range 0.005–3 V, at current densities 250 and 2500 mA·g^−1^, respectively [31].

## 3. Silicon Anodes

Silicon is cheap. Its capacity is very large: 4200 mAh·g^−1^ when lithiated to Li_4.4_Si [32]. The onset voltage potential is 0.3–0.4 V above the Li^0^/Li^+^ redox potential, which averts the safety concern of lithium deposition encountered with the graphite anode. That is why a tremendous amount of work has been done on Si-based anodes. The difficulty with Si, which has postponed its commercial use in the battery industry is the large variations of the volume during the charging/discharging process [33,34,35,36,37,38,39]: from Si to Li_4.4_Si, the volume expansion is 420% [40,41,42,43,44]. This large volume change upon cycling results in the cracking and pulverization of the Si particles and disconnection of some of the particles from the conductive carbon and from the current collector [45,46,47]. Therefore, the decrease of the size to the nano-range is mandatory to relieve the stress [48]. Only nanostructured electrodes can absorb the strains associated to the change of volume, and avoid cracking [38,49]. In addition, the decrease of the diameter of the particles upon delithiation decreases with the size of the particles, so that smaller particles can keep contact with the matrix, allowing for full extraction of the lithium. When the potential of the anode is lower than 1 V with respect to Li^0^/Li^+^, the decomposition of the organic electrolyte at the surface of the particles forms the solid-electrolyte interface (SEI). The SEI must be dense and stable to prevent further side reactions to occur. However, the large volume change makes it challenging to form a stable SEI, as it can result in a breaking of the SEI. Then, a new particle of Si can be exposed to the electrolyte, resulting in the formation of another SEI that becomes thicker and thicker upon cycling [50]. This is why the efforts on silicon anodes have been focused on the synthesis of nano-particles of different geometries, and also on the protection of the Si particles against side reactions with the electrolyte.

### 3.1. Thin Films

**Figure 2:**
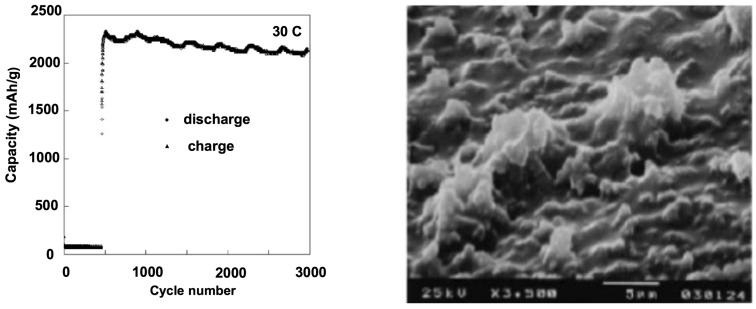
Capacity retention of 500 Å thick n-Si film during charge/discharge cycling with 30 *C* rate charge/discharge in propylene carbonate containing 1 mol·L^−1^ LiClO_4_. The scanning electron microscopy (SEM) image of the Si film is shown after 1000 cycles. Reproduced with permission from [54]. Copyright Elsevier, 2004.

To obtain good results with thin films, the silicon must be amorphous, since crystalline Si expands anisotropically upon lithiation, primarily in the <110> direction [51,52,53]. This anisotropy contributes to increasing the stress and strains in the material. In addition, it should be doped to improve the electrical conductivity. For example, a 50 nm-thick Si film n-doped with phosphor delivered a capacity 3000 mAh·g^−1^ for the case of 12 *C* test, which could be kept during 1000 cycles (Figure 2).

Additionally, in the case of the heavy load of the 30 *C* rate, the charge/discharge capacity was still over 2000 mAh·g^−1^ even after 3000 cycles [54]. This result illustrates that long cycle life up to 3000 cycles can be obtained with Si films prepared either by physical vapor deposition [54,55], or magnetron sputtering [56]. In the latter case, the thickness of the Si film deposited on Cu foil was 275 nm. On the other hand, 1 µm-thick amorphous Si film deposited on Cu foil delaminates (*i.e.*, peel off the underlying electrode) after few cycles, resulting in loss of electrical contact [57]. The thickness of the Si-film deposited on Ni foil in [54] was 500 nm. Ni develops a passivating layer that acts as a good binding agent between the substrate and the Si film due to the strong affinity of Si to oxygen. Nevertheless, even in this case, thicker films gave poorer results [54]. From these experiences, we can infer that the optimum thickness of a Si film is the order of 300 nm to avoid cracking of the film.

A new strategy of stress relaxation for Si films uses an elastomeric substrate that establishes an alternative route for new electrode design [58]. In that case, the mechanism for stress relaxation is due to the strain induced by charge/discharge cycling. The volumetric strain in Si can buckle the flat Si thin films deposited on soft substrates; such an effect releases the stress in the Si films by equilibrating the electrochemically-induced axial component and buckling induced bending component of the stress.

**Figure 3:**
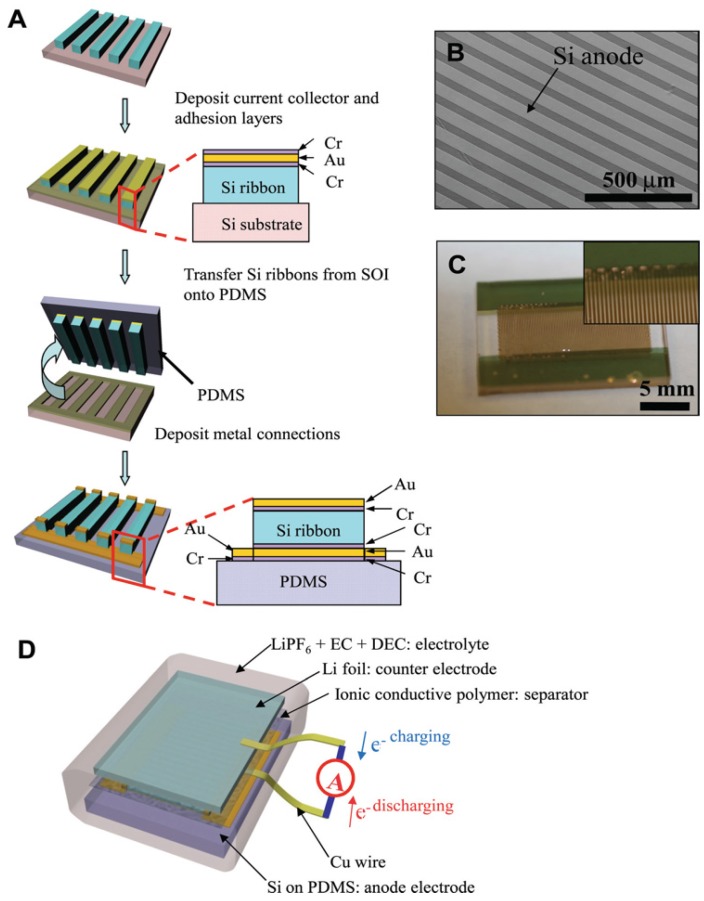
Half-cell lithium ion battery based on Si anodes, produced by microfabrication. Reproduced with permission from [58]. Copyright Wiley, 2012. (**A**) Schematic steps to fabricate the Si anodes on Poly(dimethylsiloxane) (PDMS) substrates; (**B**) A SEM image of Si anode on PDMS; (**C**) Optical images of fabricated anode before assembling; and (**D**) an illustration of the battery cell assembly.

Poly(dimethylsiloxane) (PDMS) was used as the soft substrate. The fabrication consisted in patterning the top Si (100–400 nm) of silicon-on-insulator (SOI) wafers into ribbons followed by the removal of the buried SiO_2_ layer (400 nm). Thin layers of chromium (5 nm), gold (100 nm), and chromium (5 nm) were then deposited in sequence on top of the Si ribbons, followed by oxidization of the top Cr layer. A flat PDMS substrate (300–500 μm thick) was brought into contact with the multilayer structure (Cr_2_O_3_/Au/Cr/Si). The microfabrication is shown in Figure 3.

The cell was galvanostatically charged and discharged within a voltage window of 0.005–3.000 V (*vs*. Li^0^/Li^+^) at *C*/4 rate, *i.e.*, this charge/discharge rate is calculated from the theoretical specific capacity of 4200 mAh·g^−1^ for Si (*C*/4 rate is to a current density that allows a full discharge in 4 h). Under such conditions, the cell delivered up to 4137 mAh·g^−1^ at the first cycle and at 3498 mAh·g^−1^ at the 500th cycle. Indeed, the SEM images of the buckled structures (Figure 3B), which are similar to springs, adjust the accumulated stress to avoid cracking and crumbling of the Si electrode, thus keeping the structural integrity and, thus, contributes to better cycling performance. These results show that the recent progress on thin Si films make them competitive with respect to other forms of nano-Si, such as nanowires and nanotubes, with the advantage that the synthesis process for thin films is more scalable.

### 3.2. Nanowires and Nanotubes

The silicon nanowire (Si Nw) array provides sufficient empty space between the nanowires to accommodate the change in the volume associated to the lithiation/delithiation of lithium. As an example, Figure 3 shows the electrochemical behavior of a battery using porous Si nanowires as the anode and Li metal as the current collector. Growth methods have been reviewed in [59]. The best results are obtained with wires composed of an amorphous shell deposited by chemical vapor deposition (CVD) over an as-grown crystalline core [38,60]. The main concern with the CVD method to grow Si nanostructures is the low yield. For nanowires, it is only 200–250 μg·cm^−2^ or 0.75 mg·h^−1^ [61], which greatly limits the use of this method for mass production. Nevertheless, the synthesis at the laboratory scale shows that the amorphous shell is able to prevent the initial cracking that takes place in the surface layer upon delithiation and like in the case of the thin films, n-doping with phosphor improves the electrical conductivity and, thus, the performance at high *C*-rates. Such wires delivered 3100 mAh·g^−1^ after 40 cycles at *C*/2 rate, without charging voltage limitation. The capacity retention at the 8 *C* rate was still *circa* 500 mAh·g^−1^ [62]. In this example, the nanowires were synthesized after Au-catalyzed vapor-solid-liquid (VLS) growth in a hot plate (cold wall) reactor. The temperature in the gas phase above the furnace decreases rapidly, so that after a short period of vertical growth in the stagnant layer, the wires tend to kink and curve their growth direction towards the substrate. Due to this change in growth direction, the Nws became highly entangled. The entanglement has two advantages. Firstly, the interconnection between the Nws prevents them from detaching from the substrate; and secondly, the quantity of Si is increased. In [62], it was the order of 1.2 mg per squared cm of current collector electrode, thus overcoming one of the drawbacks of using nano-materials in battery electrodes, namely the low volumetric energy density [63].

Best results are obtained when the silicon is porous, because the electrolyte can penetrate into the pores, thus increasing the effective area between Si and the electrolyte and, in addition, the pores act as buffers for the reduction of stresses during cycling. Nanoporous silicon nanowires of 5–8 µm length and with a pore size of 10 nm obtained by etching according to a process that is suitable to mass production delivered a capacity of 2400 mAh·g^−1^ with an initial coulombic efficiency of 91% and stable cycle performance [64].

Not surprising, the best result have been obtained with porous doped silicon nanowires which delivered 2000, 1600, and 1100 mAh·g^−1^ at rates 0.5 *C*, 1 *C*, 4.5 *C*, respectively, and recorded 2000 cycles with a capacity remaining above 1000 mAh·g^−1^, even achieved at 4.5 *C* [32]. Both the pore diameter and the wall thickness were about 8 nm. It should be noted that this high rate capability has been obtained at a low mass loading of 0.3 mg·cm^−2^, a recurrent problem when the nanowires are not entangled like in [63]. That is one of the reasons why the Si Nws anodes are still difficult to compete with graphite anodes. Typical commercialized graphite-based anodes can store a charge of 4 mAh·cm^−2^, because they use 50 µm thick films on the current collector. So, assuming a Si capacity of 3800 mAh·g^−1^, to obtain the same mass density Nws with their diameter in the 300 nm range have to be grown, which will limit the rate to around 3 *C* [65]. In another synthesis process, the void space of a template could be filled with the gel-like silicon precursor [66]. Mesoporous Si-carbon core-shell nanowires with a diameter of 6.5 nm prepared by this process demonstrated a first charge capacity of 3163 mAh·g^−1^ and retained a capacity at 2738 mAh·g^−1^ after 80 cycles [67].

Overall, considering the slow growth of nanowires and the low mass loading the thin Si films mentioned in the previous section, which are able to deliver stable capacity of about 1.8 mAh·cm^−2^ for 200 cycles, look more competitive. Their aging at the scale of 2000–3000 cycles, however, remains to be tested. Another problem comes from the slow growth of nanowires so that the mass production at the industrial scale is not possible yet.

**Figure 4:**
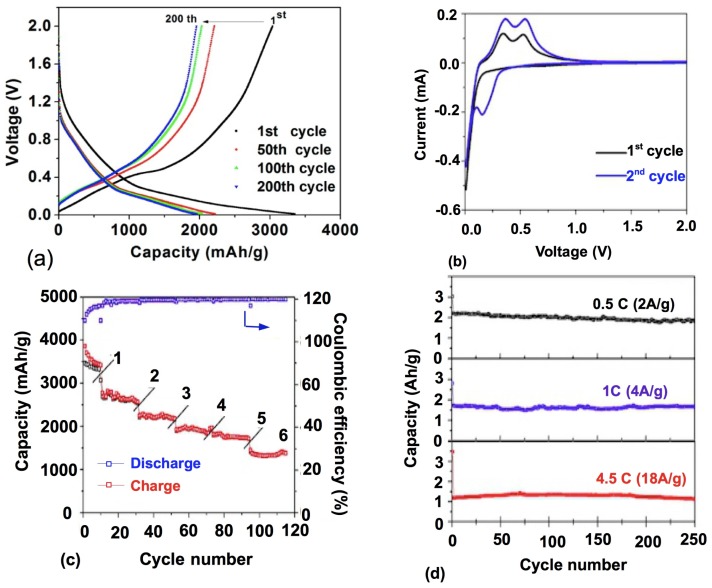

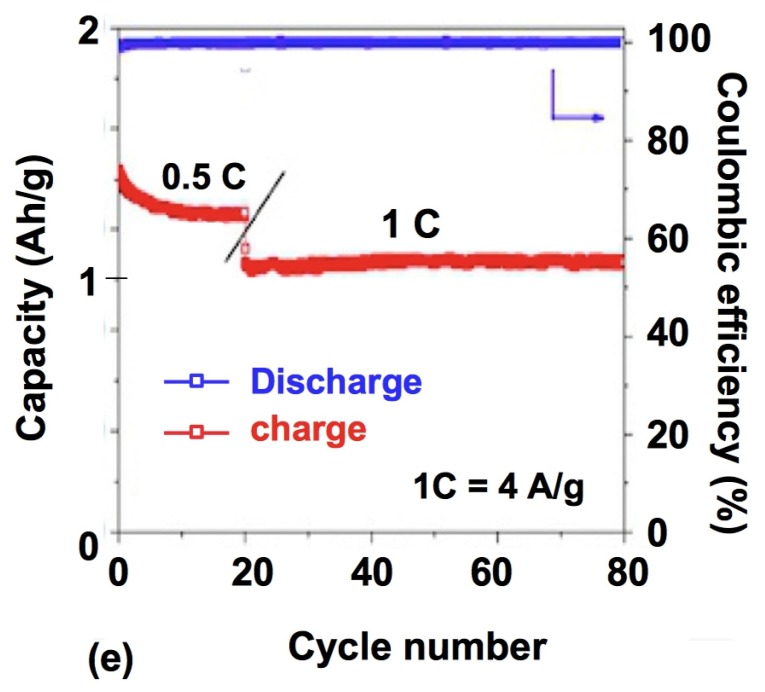
Electrochemical performance of a battery using porous silicon nanowires as the anode and lithium metal as the current collector (SEM and transmission electron microscopy (TEM) images of the nanowires are shown in Figure 5). Reproduced with permission from [32]. Copyright Wiley, 2012. Si mass loading was around 0.3 mg·cm^−2^. (**a**) Charge/discharge profile within a voltage window of 0.01−2 V *vs.* Li^0^/Li^+^ for the first cycle at a current rate of 0.4 A·g^−1^ and the 50th, 100th, and 200th cycles at 2 A·g^−1^; (**b**) cyclic voltammetry curves of porous silicon nanowire electrode for the first and second cycles using a voltage window 0.01−2 V at rate of 0.1 mV·s^−1^; (**c**) charge/discharge capacity and coulombic efficiency of porous silicon nanowire electrode at current rates of 0.6, 1.2, 2.4, 3.6, 4.8, and 9.6 A·g^−1^; (**d**) charge/discharge capacity of a porous silicon nanowire electrode at current rates of 2, 4, and 18 A·g^−1^ for 250 cycles; and (**e**) charge/discharge capacity of a porous silicon nanowire electrode at current rates of 2 A·g^−1^ (0.5 *C*) and 4 A·g^−1^ (1 *C*) with an additional 2000 cycles.

**Figure 5:**
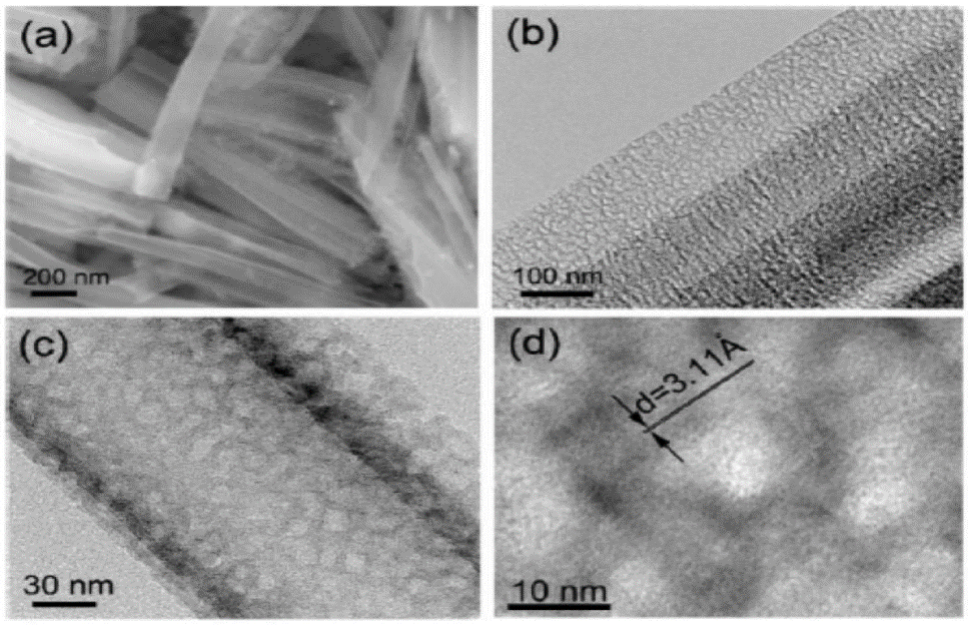
(**a**) SEM and (**b**) TEM images of porous Si nanowires; (**c**,**d**) High-resolution transmission electron microscopy (HRTEM) image of a nanowire in (**b**), leading to the results in Figure 4. Reproduced with permission from [32]. Copyright Wiley, 2012.

Nanotubes have the same advantages and drawback as nanowires. In particular, their performance is good, as it is easy to connect them electrically to the current collector [32,68,69,70] (see Figure 4 and Figure 5). Another advantage with respect to the nanowires is the very large surface area accessible to the electrolyte since lithium ions can intercalate from both the interior and the exterior of the nanotubes. As a result, the capacity 3200 mAh·g^−1^ has been reached, with capacity retention of 89% after 200 cycles at 1 C rate [70].

### 3.3. Porous Si

As already said, on one hand the porosity reduces the stress by providing additional free space for volume expansion induced by lithium-ion insertion, and another hand nanowires are not necessarily the best shape of nanostructured silicon. Different porous silicon anodes with pores of several nanometers or hollow silicon spheres with a thin shell have also been synthesized [66,71,72,73,74,75]. The pores are easily obtained by boron doping (p-type doping, while the phosphor doping mentioned earlier is n-type doping). The formation of holes in the silicon were insured by boron, which provides defective sites facilitating the etching process. This way, the structure obtained combined both properties needed to optimize the electrochemical properties: doping plus porosity. This is the procedure used, for instance, to synthesize the porous doped Nws [32] in the previous section. We have just pointed out the need for a more scalable method to prepare nano-Si. One solution has been to start with commercial silicon nanoparticles available in large quantity as starting material [76]. Such porous silicon nanoparticles after graphene wrapping delivered capacity is 2500 mAh·g^−1^ at *C*/8 rate, and 1000 mAh·g^−1^ at *C*/2 rate, and the capacity remained at 1400 mAh·g^−1^ and 1000 mAh·g^−1^ after 200 cycles at *C*/4 and *C*/2 rate, respectively. In another work, a capacity of 2000 mAh·g^−1^ for 50 cycles has been obtained with pore size of several hundred nanometers [77]. Comparable results have been obtained with porous silicon prepared by electrochemical etching with a HF etching solution [78,79]. The porosity and depth of porous Si in that case are monitored by the control of the current density and the HF concentration. The porous Si can be combined with a binder such as PAN to form a slurry self-adapting to a roll-to-roll process for mass production [80].

Porous Si can also be obtained by Si deposition into a porous template. Usually, SiO_2_ nano-spheres are used to form an opal structure. Then, porous Si can be obtained in the form of an inverse structure by filling the voids of the template. The synthesis can be carried out through CVD gas source. The results are promising [71,81,82,83], but, as we have already noted, the CVD process is not suitable for industrial scale. Instead of the CVD method, the void space of the template can be filled with the gel-like silicon precursor then heat treatment at high temperature to solidify the gel forming rigid porous silicon [66]. The product demonstrated capacity retention as high as 90% at 1 *C* rate after 100 cycles.

Instead of filling the void space of the template to get an inverse porous structure, it is also possible to convert the porous silica template into silicon directly by magnesio-thermic reduction. After carbon coating by CVD process, a three-dimensional mesoporous silicon with a high surface of 74.2 m^2^·g^−1^ obtained by this process [83]. The first reversible capacity at current density 100 mA·g^−1^ was 1956 mA h·g^−1^ (3104 mA h·g^−1^ when the mass of carbon is excluded) decreasing to 973 mA h·g^−1^ after 50 cycles.

The conducting carbon is expected to increase the electrical conductivity, since the electrons reaching the surface of a Si-particle can be driven to the current collector via the carbon, provided the carbon network percolates through the structure. The same goal can also be achieved by depositing amorphous Si directly onto electrode structure. An anode prepared in this way delivered a charge capacity of 1200 mAh·g^−1^ stable over 120 cycles [84].

### 3.4. Coated Si Nanostructures and Stabilization of the SEI

The large volume changes upon cycling imply that the SEI can be broken as the nanostructure shrinks during delithiation. This re-exposes the fresh Si surface to the electrolyte and more SEI forms, resulting in a thicker and thicker SEI film upon charge/discharge cycling and, thus, aging [85]. Stabilizing the SEI is thus crucial. This is partly done by the appropriate choice of the binder. The best one for Si-anodes is alginate which is helpful in building a deformable and stable SEI [86]. In addition, coating the silicon structure with a protective element helps in the formation of a stable SEI layer [87,88]. A thick shell is preferred to avert fracture of the nanoparticles, but a thin shell reduces the loss of weight. Therefore, careful engineering of the core and shell materials is required to obtain an optimal balance, not only to optimize (and thus control) the thickness of the shell, but also the choice of the material that must have the appropriate Young’s modulus [89,90].

**Figure 6:**
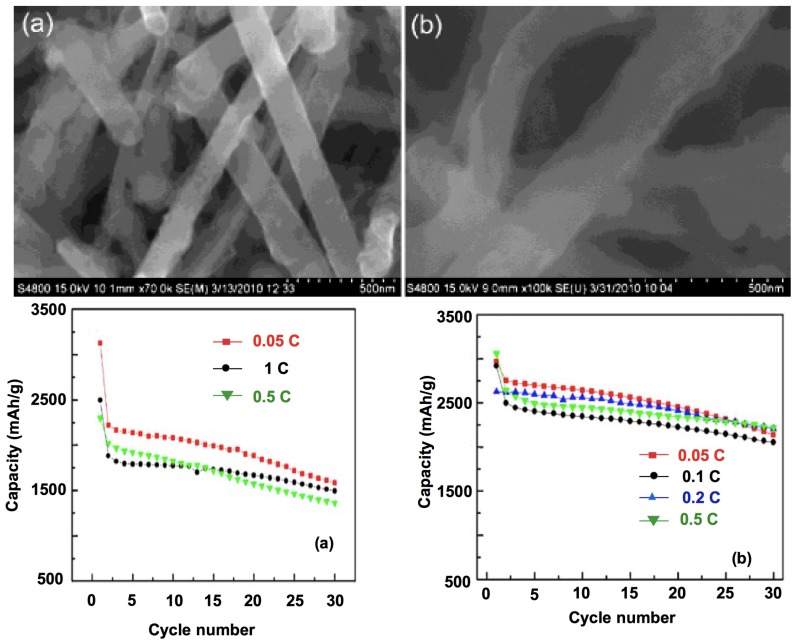
Top: SEM images of the silicon nanowires (Si Nws) (**a**) with and (**b**) without copper-coating after 100 cycles at the rate of 0.5 *C*; Bottom: capacity–cycle number curves for Si nanowires (**a**) without and (**b**) with copper-coating at different rates. Reproduced with permission from [97]. Copyright Elsevier, 2011.

The traditional process for any active material used in Li-ion batteries, whether it is used on the cathode or the anode side, is to coat the particles with conductive carbon. The same was tried with Si particles. Si-C composite particles in which silicon nanoparticles are embedded in porous carbon particles [91] and porous Si-C composite nanospheres [92] were synthesized. A successful control of the SEI growth of porous nanotubes was obtained by coating them with rigid carbon [93]. Embedding Si nanowires in a network of carbon nanotubes is also a way to improve the conductivity to improve the overall electric conductivity of the anode; moreover, the resulting anode is flexible and self-standing [94]. In [95], Si nanoparticles were completely sealed inside thin, self-supporting carbon shells, with void space in between the particles and the shells. Due to the well-defined void space, the Si particles can expand freely without breaking the outer carbon shell that stabilize the SEI on the shell surface. This yolk-shell structured Si electrode exhibits high capacity (2800 mAh·g^−1^ at *C*/10), long cycle life (1000 cycles with 74% capacity retention), and high coulombic efficiency (99.84%). With 10 nm thick carbon coating on Si nanowires of 90 nm in diameter, the first cycle coulombic efficiency was greatly enhanced from 70% (without coating) to 83%, in addition to the increased capacity from 3125 mAh·g^−1^ (without coating) to 3702 mAh·g^−1^, and cycling stability (5% capacity retention after 15 cycles) [96]. However, replacing the carbon coating with 10 nm thick Cu film can further improve the coulombic efficiency of the initial cycle up to 90.3%, and improve capacity retention up to 86% after 15 cycles [97]. We have already mentioned the results obtained on a Si-C nanocomposite in [83], but in this work too, the coating with Cu gives better results (Figure 6). These results illustrate that the traditional slurry coating method exploiting conductive carbon may not be best in the case of Si nanoparticles. To explain this result, one can invoke the fact that Cu is more conductive than carbon; it is also possible that the Cu coat is more protective and stabilizes the SEI more efficiently. In any case, this has been the motivation for trying different coats. In particular, Al-coating proved to be efficient to obtain a more stable mechanical structure of electrodes [98,99]. Si nanowires coated with *a* ≈ 100 nm layer of Ag/poly(3,4-ethylenedioxythiophene) (PEDOT) exhibited an improvement of the capacity retention from 30% after few cycles, to 80% after 100 cycles, with respect to the same wires before coating [100]. These coats have in common the fact that they are metallic, *i.e.,* conducting. However, Al_2_O_3_ coatings (<10 nm) obtained by ALD have been tested successfully on Si thin films [101,102] and Si Nws [103]. Upon the first lithiation, Al_2_O_3_ transforms into an Al-Li-O glass [104], which is a good ionic conductor and an electronic insulator, thus exhibiting the attributes of a good SEI substitute. Indeed, the Al_2_O_3_ coating resulted in a 45% increase of the anode lifetime, and 1280 cycles at 1 *C* have been obtained with Al_2_O_3_ coated Si Nws [103]. Double walled Si nanotubes have been obtained by coating the Si nanotube with SiO*_x_* [105]. This coat is rigid enough and mechanically strong, so that it can successfully prevent the Si from expanding outward during lithiation, while still allowing lithium ions to pass through. As a result, the SiO*_x_*-coated Si nanotubes in [93] demonstrated a long cycle life (6000 cycles with 88% capacity retention), high capacity (2970 mAh·g^−1^ at *C*/5; 1000 mAh·g^−1^ at 12 *C*), and fast charging/discharging rates (up to 20 *C*). This result shows that it is now possible to obtain Si anodes with high capacity and good capacity retention for thousands of cycles at the laboratory scale.

However, the cost of the synthesis processes is still a limiting factor for large size battery applications. The process described in [105] using vertically aligned carbon nanotubes (VACNTs) uniformly coated with Si and a thin C surface layer is a scalable method to produce ultra-thick, yet highly conductive and stable, Li-ion battery electrodes. Such an anode demonstrated very good stability for over 250 cycles and high specific capacity approaching theoretical limits (4200 mAh·g^−1^). Recently, a robust method to prepare gram-scale Si NTs has been reported using nanorod-like nickel-hydrazine complexes as templates [106]. These different results show that the efforts currently done to find scalable synthesis processes without altering the electrochemical performance of Si anodes open the route to their mass production in the near future.

## 4. Li_4_Ti_5_O_12_

The Li_4_Ti_5_O_12_ (LTO) spinel is now considered as a viable anode for Li-ion batteries. It exhibits excellent Li-ion reversibility according to a two-phase reaction maintaining the constant voltage at 1.55 V *vs.* Li^0^/Li^+^. Of course, this rather high voltage reduces by 1.2 V the operational potential with a cell equipped with a classical carbon anode, and this means a reduction of energy density. On the other hand, this drawback is compensated by other advantages. The SEI forms on the anodes, whether they are carbon, graphite, or metal oxides, as soon as the voltage is lower than 1 V during Li insertion. In that case, the formation of the SEI is controlled by the reduction of the solvents present in the electrolyte, namely ethylene carbonate (EC) and diethyl carbonate (DEC), aided by the presence of the Li salt (LiPF_6_).

The reaction at 1.55 V implies that no SEI is formed at the surface of LTO. Not only does it avoid the safety problems linked to the SEI with other anodes, but it also makes it possible to get rid of EC to use other electrolytes allowing the fabrication of batteries working at high temperature 80 °C [107], or subzero temperature [108], which is not possible with other Li-ion batteries. It is cheap, very safe, environmental friendly, and has a remarkable structural stability. In addition, Li cycling involves very little change in the cubic lattice parameter so that LTO is a “zero strain” material, resulting in a much longer cycling life. LTO is, thus, ideally suited as an anode material. That is why a large number of papers have been devoted to this material, reviewed in [2,109,110,111].

**Figure 7:**
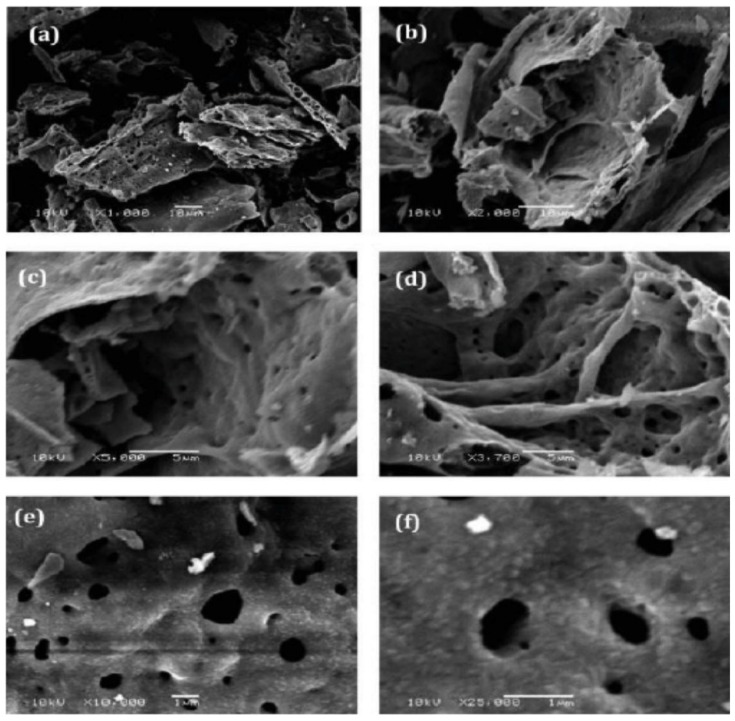
SEM images for nanocrystalline Li_4_Ti_5_O_12_ at different magnifications synthesized by combustion method. Reproduced with permission from [110]. Copyright American Chemical Society, 2013.

**Figure 8:**
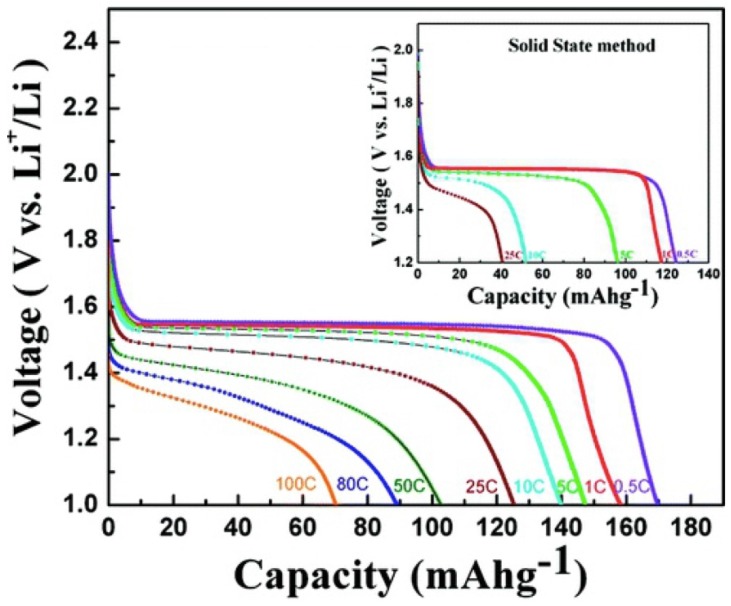
Capacity-voltage profile for nanocrystalline Li_4_Ti_5_O_12_ (SEM images in Figure 7) synthesized by the combustion method at different *C*-rates. Inset shows capacity voltage profile for bulk Li_4_Ti_5_O_12_ prepared by the solid-state method. Reproduced with permission from [108]. Copyright Elsevier, 2014.

Like in the case of Si, however, the electrical conductivity of LTO is small, and this problem can be solved owing to the same remedy: use nano-structured LTO, eventually carbon coated, and preferentially porous, to increase the effective surface area with the electrolyte. For instance, porous LTO crystallites with a surface area of 12 m^2^·g^−1^, of varying size between 20 and 50 nm, delivered a capacity value close to the theoretical value of 175 mAh·g^−1^ at *C*/2 rate, and 140 and 70 mAh·g^−1^ at 10 *C* and 100 *C* discharge rates, respectively, without significant aging over 100 cycles as shown in Figure 7 and Figure 8 [112]. Mesoporous nest-like LTO with large surface area 219.2 m^2^·g^−1^ delivered 113.6 mAh·g^−1^ were obtained at 57 *C* [113]. 10 nm-thick nano flower-like LTO delivered 118 mAh·g^−1^ at 30 *C* [114]. These examples show that LTO nanoparticles of different morphologies are performing even at a high *C*-rate.

To obtain even better results, nano-LTO (nano-rods, hollow spheres, nanoparticles) has been carbon-coated [107,115,116]. C-LTO nanoparticles with 90 nm in size showed a capacity of 166 mAh·g^−1^ at *C*/24, remaining larger than 150 mAh·g^−1^ at 40 *C*-rate [107]. LTO consisting in 0.5–1 µm sized porous microspheres composed of 11 nm-sized crystallites (pore diameter 4.3 nm) delivered 158 and 100 mAh·g^−1^ at 1 *C* and 50 *C*, respectively without noticeable capacity fading [117]. LTO nano-platelets of size 10–20 nm uniformly dispersed on reduced graphite oxide (RGO) to form a nano-hybrid composite delivering a capacity of 101 mAh·g^−1^ at 100 *C* rate [118].

The tests on the electrodes of Li-ion batteries are always made on half-cells, *i.e.*, with lithium metal as counter-electrode. Due to advantages of LTO over graphite anodes, full lithium-ion batteries with LTO anodes and different cathodes have also been tested. LTO has first been associated with LiMn_2_O_4_ spinel [119,120]. High-power capabilities of nano-LTO/LiMn_2_O_4_ batteries, using 20 nm LTO with fast charge capability up to 80 *C* over 1000 cycles has been reported [121]. Nano-LTO/LiMn_2_O_4_ cells with micrometer size (∼0.5–2 μm) secondary particles composed of nanosize (≤10 nm) primary particles LTO as the anode and spherical particles of LiMn_2_O_4_ as the cathode have also been constructed [122]. Nearly 100% capacity retention was reported over 1000 cycles at 5 *C* rate at 55 °C together with excellent performance at low temperature (−30 °C). These performances make such a cell suitable for HEVs and energy-storage for electric grids. LTO has also been associated with lamellar compounds as the counter-electrode [120,123,124,125].

Outstanding results have been obtained with LiFePO_4_ as the cathode. Capacities of 155 and 122 mA h·g^−1^ at 0.1 *C* and 5 *C* rates with nanoscale LTO/LiFePO_4_ [126]. The size of the LTO particles ranged from 50 to 200 nm, while the LiFePO_4_ particles of size 50–100 nm were coated with 2–4 nm thick carbon. A safe and fast-charging LIB with long shelf life for power applications used LTO (150 nm particle size) as anode and 2 wt % carbon- coated nano-LiFePO_4_ (particle size: 25 nm) as cathode [127]. Full capacity after 20000 cycles performed at charge rate of 10 *C* and discharge rate of 5 *C*, and retained 95% of the capacity after 30000 cycles at charge rate 15 *C* and discharge rate 1 *C*, both at 100% depth of discharge) and 100% state of charge. Even higher powers have been obtained by coating these LTO particles with carbon [105].

## 5. Conclusions

The nanotechnology and its application to the synthesis of nano-structured electrodes is the key of the progress that has been currently made on lithium-ion batteries. In the case of positive electrodes, this nanotechnology opens the route to the replacement of the graphite. Different batteries have been tested, especially employing Nano-sized Li_4_T_5_O_12_. They showed outstanding performance regarding the *C*-rate capability, cycling stability, calendar life, pulse-power characteristics, and abuse tolerance. Such anodes should now be developed over the next years for many applications. Silicon is very promising for its very large theoretical capacity, some scalable synthesis processes opening the route to prepare nano-Si anodes at a reasonable price. The few examples given in this review also illustrates the large variety of morphologies that can be used, which combines with the large variety of oxides of transition metals, making the research on the materials science active and promising for years to come.
